# Single-fiber reflectance spectroscopy system using unmodified clinical laser fibers for tissue optical property recovery for photodynamic therapy treatment planning

**DOI:** 10.1117/1.JBO.31.9.097001

**Published:** 2026-09-02

**Authors:** Md Nafiz Hannan, Timothy M. Baran

**Affiliations:** a University of Rochester, Department of Physics and Astronomy, Rochester, New York, United States; b Department of Imaging Sciences, University of Rochester Medical Center, Rochester, New York, United States; c University of Rochester, Department of Biomedical Engineering, Rochester, New York, United States; d University of Rochester, The Institute of Optics, Rochester, New York, United States

**Keywords:** single-fiber reflectance spectroscopy, clinical optical fiber, Monte Carlo simulation, optical property estimation, inverse problem, photodynamic therapy

## Abstract

**Significance:**

Estimation of tissue optical properties in workflow- and resource-constrained clinical settings is required to support photodynamic therapy (PDT) treatment planning, yet large-scale clinical translation remains limited by spectroscopy probe cost, complexity, and the need for probe-specific inverse models. Single-fiber reflectance (SFR) spectroscopy offers a simple and low-cost option, but previous implementations relied on customized angle-polished fibers.

**Aim:**

The objective was to develop a proof-of-concept SFR spectroscopy system employing an unmodified, United States Food and Drug Administration (FDA)-approved clinical optical fiber for quantitative optical property extraction.

**Approach:**

A semi-empirical photon pathlength model was used to recover the absorption spectra $\mu_{a}(\lambda )$ and reduced scattering spectra $\mu_{s}^{\prime }(\lambda )$ from tissue-mimicking phantoms containing methylene blue (MB) as the absorber and Intralipid-20% as the scatterer.

**Results:**

Optical property retrieval from measurements acquired using three nominally identical fibers demonstrated clinically sufficient accuracy across fibers without requiring model adaptation. Across all fibers, MB concentration $\left(C_{\mathrm{MB}}\right)$ was recovered with a root mean square error (RMSE) of 0.6 to 0.7  μM (p=0.88 among fibers), while $\mu_{s}^{\prime }(665\;\mathrm{nm})$ was recovered with an RMSE of 1.5 to $2.2\;{\mathrm{cm}}^{-1}$ (p=0.68 among fibers).

**Conclusions:**

We demonstrate the potential of SFR spectroscopy using a standard clinical optical fiber as a scalable and clinically useful tool for tissue optical property estimation to support PDT treatment planning.

## 1 Introduction

Photodynamic therapy (PDT) is a light-based treatment modality in which a photosensitizer is excited by illumination at an appropriate wavelength.^1^ In the presence of molecular oxygen, this excitation generates cytotoxic reactive oxygen species, leading to cellular death. Due to the strong anti-microbial effects of PDT, it has been successfully applied to treat various microbial infections.^2^^,^^3^ We are particularly interested in the application of anti-microbial PDT for disinfecting abdominopelvic abscesses.^4^ Safe and effective PDT requires proper treatment planning to deliver an efficacious light dose while sparing surrounding healthy tissue.^5^ Determining optimal light delivery requires accurate knowledge of the optical properties of the target tissue. Hence, accurate measurement of tissue optical properties is crucial for designing safe and efficacious treatment plans.^6^^–^^9^ However, previous approaches have typically required separate devices for optical property extraction, increasing cost and complexity.

The optical properties of tissue are commonly characterized by the absorption coefficient $\mu_{a}$, the scattering coefficient $\mu_{s}$, and the scattering phase function p(θ).^10^ If photons undergo many scattering events before detection, tissue scattering can be described by the reduced scattering coefficient, $\mu_{s}^{\prime }=\mu_{s}(1-g)$, where g is the scattering anisotropy. For our treatment planning purposes, $\mu_{a}$ and $\mu_{s}^{\prime }$ are the most relevant parameters.^6^^,^^11^ To that end, we previously constructed and validated a diffuse reflectance spectroscopy (DRS) system^12^ capable of extracting tissue optical properties using a fiber-optic probe placed in contact with the abscess wall, which was employed in our phase 1 clinical trial for disinfection of abdominopelvic abscesses.^4^ Although this device accurately estimated abscess wall optical properties,^13^ it had substantial translational limitations for large-scale, multi-center clinical deployment. The custom-manufactured, multi-fiber probes increased cost and lead time, reduced robustness, and required careful handling. As these probes were not built to withstand the high laser power (>1  W) required for PDT treatment, separate fiber-optic probes were required for spectroscopy and treatment light delivery. In addition, the optical property retrieval model was probe-specific,^14^ necessitating resource-intensive calibration and validation for each new probe. Finally, the requirement for probe sterilization before and after each use further limits the feasibility for routine clinical implementation.

**Fig. 1:**
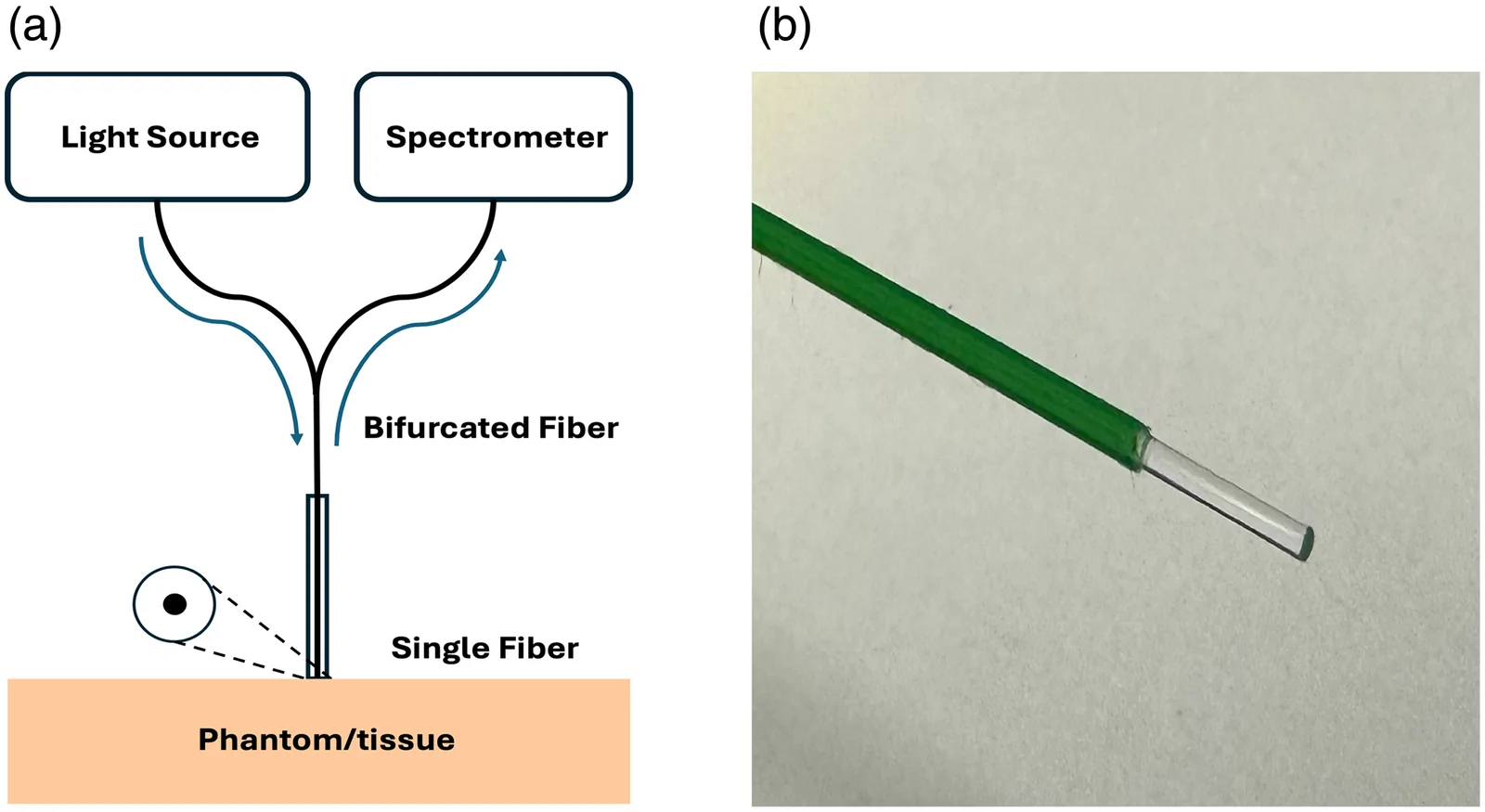
(a) Schematic of the single-fiber spectroscopy system. (b) Distal end of the flat-cleaved optical fiber.

Our aim is to further investigate PDT for the treatment of abscess cavities through advanced clinical studies, paving the way for widespread clinical adoption. Achieving this objective requires overcoming several key translational barriers, including cost, complexity, and clinical feasibility. In this regard, single-fiber reflectance (SFR) spectroscopy offers an attractive solution due to its simplicity, scalability, and ease of clinical integration. SFR spectroscopy employs a single optical fiber for both illumination and detection (Fig. 1). Upon direct contact between the fiber and tissue, measured reflectance enables quantitative extraction of tissue optical properties. Compared with multi-fiber DRS systems, the estimation accuracy of SFR spectroscopy is typically diminished.^15^ However, to realize our longer-term goal of multi-center clinical trials, we prioritize cost reduction, clinical deployability, and minimal disruption of clinical workflow over maximizing estimation performance.

The clinical utility of SFR spectroscopy has been demonstrated across multiple applications, including tissue optical property estimation,^16^^,^^17^ differentiation of normal and metastatic lymph nodes in lung cancer staging biopsies,^18^ monitoring of cerebral hemodynamics during deep brain stimulation,^19^ and enhanced endoscopic surveillance in patients with Barrett’s esophagus.^20^ However, these prior studies required modifications to the optical fiber, including the use of angle-polished fibers or multiple individual fibers with different diameters. For our clinical application, the use of an angle-polished fiber poses an increased risk of abscess perforation due to its sharp tip, potentially leading to leakage of infected fluid from the abscess cavity, which can result in sepsis and other life-threatening conditions. Using multiple single fibers with different diameters can enable improved optical property recovery performance compared with the use of a single fiber.^17^^,^^21^ However, they are not well-suited for our clinical application due to their cost, increased outside diameter, and lack of United States Food and Drug Administration (FDA)-approved commercial availability.

Here, we employ an FDA-approved, unmodified flat-cleaved single optical fiber for optical property recovery. Compared with custom or angle-polished fibers, the use of this fiber offers several translational advantages: it is safer for clinical use, compatible with smaller catheter channels, less expensive, and commercially available. Importantly, because the fiber-to-fiber variability is small, the same optical property extraction model can be repeatedly used across nominally identical fibers without the need for fiber-specific calibration. As the fiber already has FDA clearance for clinical use and is delivered in a sterile, single-use package, it can be clinically deployed without additional sterilization and regulatory issues. Critically, the same fiber can also be employed for both spectroscopy and PDT treatment light delivery. In our intended clinical scenario, for each patient, the clinician would remove a sterile fiber from its packaging, perform SFR spectroscopy, compute PDT treatment parameters, deliver treatment light using the same fiber, and discard after use. The ability to perform both spectroscopy and PDT with a sterile, single-use fiber for each new patient, without requiring time-consuming model development or calibration, substantially simplifies clinical implementation. Together, these factors make SFR spectroscopy particularly suitable for enabling our envisioned large-scale clinical translation of optical-property-informed PDT for abdominopelvic abscess disinfection.

In this work, we developed and validated an SFR spectroscopy system using an unmodified clinical fiber for quantitative tissue optical property estimation to support PDT treatment planning. We demonstrate that a semi-empirical model of reflectance enables optical property recovery with clinically sufficient accuracy in phantoms and achieves consistent performance across nominally identical fibers without the need for model adaptation.

## 2 Methods

### 2.1 Fiber Optic and Spectroscopy System

In this continuous-wave SFR spectroscopy system [Fig. 1(a)], a flat-cleaved, multimode clinical optical fiber (Flexiva 550, Boston Scientific, Marlborough, Massachusetts, United States) [Fig. 1(b)] was used for light delivery and collection. This fiber had a 550-μm core and a 1-mm outer diameter. Broadband light (500 to 800 nm) from a halogen lamp (AvaLight-Hal-S, Avantes, Lafayette, Colorado, United States) was coupled into one arm of a bifurcated fiber (BIF400-2-VIS-NIR, Ocean Optics, Orlando, Florida, United States) and guided into the optical fiber, which was connected using a butt coupler. After interacting with tissue, reflected light—comprising specular reflection at the surface as well as sub-diffuse and diffuse reflectance within the tissue—was collected by the same fiber and transmitted through the other arm of the bifurcated fiber to a spectrometer (Compass X, B&W Tek, Plainsboro, New Jersey, United States) for acquisition. A neutral density filter (FBR-ND10, Newport Corporation, Irvine, California, United States) was placed in front of the spectrometer to prevent detector saturation.

### 2.2 Monte Carlo Simulations

The graphics processing unit (GPU)-accelerated CUDAMCML framework^22^ was modified to incorporate a Monte Carlo (MC) model of the flat-cleaved fiber. Simulations were used to investigate how tissue optical properties ($\mu_{a}$, $\mu_{s}$, and g), together with fiber-related parameters such as fiber diameter and numerical aperture (NA), influence the SFR signal. The model was also used to examine photon pathlength distributions and to develop a model for the mean photon pathlength as a function of optical properties.

MC simulations were conducted with the optical fiber oriented normal to the tissue surface, with the distal face placed in direct contact with the medium to match the experimental configuration. Photon packets were emitted from the source fiber with a uniform angular distribution within the NA. Upon reaching the fiber–tissue boundary, Fresnel coefficients calculated from the refractive indices of the fiber and tissue were used to determine photon transmission and reflection on a per-packet basis. The reflected component of the photon weight was recorded as specular reflectance, while photon packets transmitted into the tissue were allowed to propagate and undergo absorption and scattering. Photon packets that later re-entered the probe within the fiber core diameter and acceptance NA were counted as detected reflectance, comprising both sub-diffuse (singly scattered) and diffuse (multiply scattered) contributions. The photon weights at the time of re-entry were recorded and summed to compute single-fiber reflectance $R_{\mathrm{SF}}^{\mathrm{MC}}$. For photon packets incident on the air–tissue interface outside the fiber diameter, Fresnel reflections were computed using the refractive indices of air and tissue.

Tissue was modeled as a semi-infinite medium with homogeneous optical properties and a refractive index of 1.37. The fiber was modeled with a core diameter of 550  μm, an outer diameter of 1 mm, a core refractive index of 1.46, and an NA of 0.37. Simulations were performed under the following conditions: $\mu_{a}$ ranged from 0.001 to $5.05\;{\mathrm{cm}}^{-1}$, $\mu_{s}$ ranged from 1 to $400\;{\mathrm{cm}}^{-1}$, and the scattering phase function was represented by the Henyey–Greenstein phase function with anisotropy factor g∈{0.6,0.7,0.8,0.9}. The corresponding $\mu_{s}^{\prime }=\mu_{s}(1-g)$ values were also calculated, and $\mu_{s}^{\prime }$ values up to $40\;{\mathrm{cm}}^{-1}$ were considered. The mean photon pathlength $\left\langle L_{\mathrm{SF}}^{\mathrm{MC}}\right\rangle$ was determined using the following equation:

$$\langle L_{\mathrm{SF}}^{\mathrm{MC}}\rangle =\frac{\overset{N}{\underset{i=1}{\sum }}w_{i}L_{i}}{\overset{N}{\underset{i=1}{\sum }}w_{i}},$$ (1)

where N is the number of detected photon packets, $w_{i}$ is the detected weight of the i’th photon packet, and $L_{i}$ is the corresponding photon packet pathlength. Two four-dimensional libraries were constructed over the parameter space $\left(\mu_{a},\mu_{s}^{\prime },\;\text{and}\;g\right)$ with corresponding values of $R_{\mathrm{SF}}^{\mathrm{MC}}$ and $\left\langle L_{\mathrm{SF}}^{\mathrm{MC}}\right\rangle$. Each simulation launched $10^{8}$ photon packets, and computations were performed on an NVIDIA Quadro RTX6000 GPU with 24 GB of memory (NVIDIA Corporation, Santa Clara, California, United States).

### 2.3 Data Collection and Calibration

Extraction of optical properties from SFR measurements requires calibration of the collected spectra to a power-normalized absolute scale. In addition, the calibration procedure must incorporate corrections for wavelength-dependent effects and internal reflections, as well as eliminate the specular reflection from the fiber–sample interface. Calibration was performed using the following equation adapted from Zhang et al.,^23^ where calculated Fresnel reflection at the fiber–air interface $R_{\text{air}}$ was used as the reference:

$$R_{\text{sample}}(\lambda )=\frac{R_{\text{air}}}{P_{\mathrm{tot}}}\cdot \frac{I_{\text{sample}}(\lambda )-I_{\text{water}}(\lambda )}{I_{\mathrm{air}}(\lambda )-I_{\text{fiber match}}(\lambda )}.$$ (2)

Calibration involved three reference measurements: $I_{\text{air}}(\lambda )$, measured in air; $I_{\text{water}}(\lambda )$, measured in water; and $I_{\text{fiber match}}(\lambda )$, measured in glycerin. The calibrated signal was then divided by $P_{\mathrm{tot}}$, the total optical power at a reference wavelength (650 nm). All spectra were corrected for dark current by subtracting a spectrum acquired with the lamp output disabled.

### 2.4 Phantom Preparation

For phantom experiments, methylene blue (MB, Akorn, Inc., Lake Forest, Illinois, United States) was chosen as a representative absorber, and Intralipid-20% (Fresenius Kabi AG, Bad Homburg, Germany) was chosen as a representative scattering medium, with deionized water used as the solvent. For each phantom, the required volumes of MB and Intralipid were calculated to achieve the desired optical properties and added to 50 mL of deionized water in a black spray-painted container (Rust-Oleum Flat Black, Vernon Hills, Illinois, United States).

To calculate the required volumes of Intralipid and MB needed to achieve the desired phantom optical properties, the absorption spectra of MB, the scattering spectra of Intralipid, and the scattering anisotropy g of Intralipid were required. The absorption spectra $\mu_{a}(\lambda )$ of MB and the scattering spectra $\mu_{s}(\lambda )$ of Intralipid were measured using a commercial spectrophotometer as we have reported previously (Cary 50, Varian, Santa Clara, California, United States).^12^^,^^24^ The scattering anisotropy g of the Intralipid stock was determined experimentally using a separate set of phantoms. A total of 30 phantoms were prepared with $\mu_{s}$ of 25, 37.5, 62.5, 75, and $125\;{\mathrm{cm}}^{-1}$ at 665 nm, each with $\mu_{a}$ of 0.05, 0.1, 0.25, 0.5, 0.75, and $1.0\;{\mathrm{cm}}^{-1}$ at 665 nm. The data from these phantoms were collected using our previously described DRS system.^12^ The scattering anisotropy g of the Intralipid stock was then calculated using the previously reported methodology.^14^ These phantoms were only used for determination of scattering anisotropy for the Intralipid stock and were not used for SFR spectroscopy.

To evaluate the SFR system performance, three validation phantom datasets were collected using three separate single optical fibers (fibers 1, 2, and 3), with an independent set of phantoms prepared for each fiber, denoted $V_{F1}$, $V_{F2}$, and $V_{F3}$, respectively. These validation datasets spanned $\mu_{s}^{\prime }$ values of 5, 10, 15, 20, 25, 30, and $35\;{\mathrm{cm}}^{-1}$ at 665 nm, each with $\mu_{a}$ values of 0.1, 0.25, 0.5, 0.75, and $1.0\;{\mathrm{cm}}^{-1}$ at 665 nm, resulting in 35 phantoms per fiber and a total of 105 phantoms across the 3 fibers. The validation range was selected to encompass optical property values representative of reported tissue optical properties.^13^

The experimental and MC-derived SFR signals were on different scales due to small mismatches between the experimental phantom conditions and the MC model, such as coupling and the presence of additional interfaces. To calibrate the experimental measurements to the MC scale, two additional phantom measurements were acquired using fiber 1, consisting of $\mu_{s}^{\prime }$ values of 3 and $35\;{\mathrm{cm}}^{-1}$ at 665 nm, each with $\mu_{a}$ values of $0.05\;{\mathrm{cm}}^{-1}$ at 665 nm. These calibration phantoms were not used to evaluate system performance. A single global calibration factor, r, was determined using these phantoms with known optical properties by minimizing the difference between the experimentally measured and MC-simulated reflectance spectra. This calibration factor was not re-estimated for individual phantoms or fibers and was applied unchanged to all validation phantoms ($V_{F1}$, $V_{F2}$, and $V_{F3}$) to rescale the experimental measurements onto the MC scale prior to optical property extraction.

Prior to phantom measurements, the lamp was allowed to reach steady state for 15 min. All measurements were performed in a semi-infinite geometry, with the fiber placed in direct contact with the medium. A sequence of calibration measurements was acquired, including measurements in air $I_{\text{air}}(\lambda )$, deionized water $I_{\text{water}}(\lambda )$, and glycerin $I_{\text{fiber match}}(\lambda )$, which has a refractive index of 1.47 at 650 nm. The total optical power emitted from the fiber at 650 nm $\left(P_{\mathrm{tot}}\right)$ was measured using a 6-in. integrating sphere (Labsphere, North Sutton, New Hampshire, United States). These measurements were acquired once for each phantom experiment. Each experiment consisted of a phantom with a single $\mu_{s}^{\prime }$ value, with MB added sequentially to achieve the target $\mu_{a}$ values specified above. Following calibration, the fiber was placed in contact with the phantom to acquire the reflectance measurement. For each calibration and reflectance measurement, a corresponding dark measurement was recorded and subtracted. Individual spectral acquisitions used variable integration times up to 200 ms such that the peak signal corresponded to ∼66% of the maximum value allowed by the spectrometer, and a complete measurement sequence, including calibration and reflectance measurements for a given phantom condition, was completed in ∼3  min.

### 2.5 Semi-Empirical Model and Recovery of Optical Properties

A semi-empirical model developed by Kanick et al.^25^ was used to relate the detected SFR spectrum $R_{\mathrm{SF}}$ to the underlying optical properties $\left(\mu_{a},\mu_{s}^{\prime },\;\text{and}\;g\right)$. In this formulation, $R_{\mathrm{SF}}$ is expressed as the product of an absorption-free scattering baseline term $R_{0}$ and an absorption-dependent exponential term involving the mean photon pathlength $\left\langle L_{\mathrm{SF}}\right\rangle$

$$R_{\mathrm{SF}}(\lambda )=R_{0}(\lambda )e^{-\mu_{a}(\lambda )\langle L_{\mathrm{SF}}\rangle }.$$ (3)

This factorization decouples scattering-dominated spectral scaling (captured by $R_{0}$) from absorption-induced attenuation, enabling joint estimation of scattering and absorber content from a single broadband spectrum.

The mean photon pathlength $\left\langle L_{\mathrm{SF}}\right\rangle$ was modeled as a function of $\left(\mu_{a},\mu_{s}^{\prime },\;\text{and}\;g\right)$ using an empirical expression adapted from Kanick et al.^25^

$$\langle L_{\mathrm{SF}}^{\text{model}}\rangle =\frac{(p_{1}+p_{2}\;g)d_{\text{fiber}}}{\left(\mu_{s}^{\prime }d_{\text{fiber}}{)}^{p_{3}}+[p_{4}+p_{5}(\mu_{a}d_{\text{fiber}}{)}^{p_{6}}\right]},$$ (4)

where $d_{\text{fiber}}$ is the fiber diameter, g is the scattering anisotropy, and the parameters $\left\{p_{1},\ldots ,p_{6}\right\}$ were obtained by minimizing the residual error between $\left\langle L_{\mathrm{SF}}^{\text{model}}\right\rangle$ and the MC-derived pathlengths $\left\langle L_{\mathrm{SF}}^{\mathrm{MC}}\right\rangle$. The values of these parameters, determined from the MC model of the single fiber described above, were fixed at this stage and were not re-determined for individual fibers or phantom conditions. This mean photon pathlength model was used unchanged for all validation phantoms.

Independent recovery of $\mu_{a}(\lambda )$, $\mu_{s}^{\prime }(\lambda )$, and g(λ) at each wavelength from a single SFR spectrum is not possible, as the resulting inverse problem is under-determined. Hence, to ensure robust and reproducible extraction of optical properties from SFR, spectral fitting was performed using known absorber and scatterer spectral shapes. The absorption-free baseline $R_{0}$ and the optical properties $\left(\mu_{a}\;\text{and}\;\mu_{s}^{\prime }\right)$ were parameterized to reduce the number of free parameters. $R_{0}(\lambda )$ was parameterized as the sum of two power law terms representing singly (sub-diffusive) and multiply scattered photon (diffusive) contributions, as shown in Eq. (5). The reduced scattering coefficient $\mu_{s}^{\prime }(\lambda )$ and absorption coefficient $\mu_{a}(\lambda )$ were modeled as

$$R_{0}(\lambda )=a_{1}{\left(\frac{\lambda }{\lambda_{0}}\right)}^{a_{2}}+a_{3}{\left(\frac{\lambda }{\lambda_{0}}\right)}^{a_{4}},\;a_{1},a_{2},a_{3}>0,a_{4}<0,$$ (5)

$$\mu_{s}^{\prime }(\lambda )=b_{1}{\left(\frac{\lambda }{\lambda_{0}}\right)}^{b_{2}},\;b_{1}:\;\text{scattering scale},\;b_{2}:\;\text{scattering exponent},$$ (6)

$$\mu_{a}(\lambda )=C_{\mathrm{MB}}A(\lambda ),\;\begin{array}{l} C_{\mathrm{MB}}:\;\mathrm{MB}\;\text{concentration},\\ A(\lambda ):\;\mathrm{MB}\;\text{absorption basis}, \end{array}$$ (7)

where λ and $\lambda_{0}$ denote the wavelength and normalization wavelength (665 nm), respectively.

As the quantities of interest were $\mu_{a}$ and $\mu_{s}^{\prime }$, the extraction algorithm was designed to emphasize the corresponding model parameters $\left(b_{1},b_{2},\;\text{and}\;C_{\mathrm{MB}}\right)$. Variations in g within the physiologically relevant range (0.6 to 0.9) did not substantially affect the recovered results (<5% for $C_{\mathrm{MB}}$); therefore, g was fixed at 0.9. Combined with the previous parameterization, this reduced the total number of fitting parameters to seven $\left(a_{1},a_{2},a_{3},a_{4},b_{1},b_{2},\;\text{and}\;C_{\mathrm{MB}}\right)$. A two-stage, constrained nonlinear optimization was then employed to fit the model to the experimental SFR spectra. Stage I estimated $\left(a_{1},a_{2},a_{3},a_{4},\;\text{and}\;C_{\mathrm{MB}}\right)$, while stage II refined $\left(b_{1},b_{2},\;\text{and}\;C_{\mathrm{MB}}\right)$ corresponding to $\left(\mu_{a}\;\text{and}\;\mu_{s}^{\prime }\right)$. This sequential strategy limited the number of simultaneously optimized parameters, thereby reducing parameter cross-talk and improving stability. All fitting parameters were normalized to ensure robust convergence.

In stage I, the mean photon pathlength was fixed to a constant equal to the average of the MC-derived mean pathlengths computed across all simulated optical property combinations, $\langle L_{\mathrm{SF}}\rangle =0.2\;\mathrm{cm}$. Fixing $\left\langle L_{\mathrm{SF}}\right\rangle$ reduced the dimensionality of the optimization problem, such that only five free parameters ($a_{1},a_{2},a_{3},a_{4}$, and $C_{\mathrm{MB}}$) were estimated in stage I. Because $R_{0}$ scales linearly with $\mu_{s}^{\prime }$, $R_{0,665}=a_{1}+a_{3}$ was used to initialize the estimation of $\mu_{s,665}^{\prime }=b_{1}$ in the subsequent stage.

In stage II, the full pathlength model (Eq. 4) was used, and $\left(b_{1},b_{2},\;\text{and}\;C_{\mathrm{MB}}\right)$ were the only free parameters. They were estimated via constrained nonlinear optimization, with biologically relevant bounds ($b_{1}\in [0,50]\;{\mathrm{cm}}^{-1}$, $b_{2}\in [-2,2]$, and $C_{\mathrm{MB}}\in [0,50]\;\mu M$). All calculations and analyses were performed in MATLAB (R2023b, The MathWorks, Inc., Natick, Massachusetts, United States).

### 2.6 Statistical Analysis

The measured values are reported as mean ± standard deviation. The accuracy of the recovered optical properties was quantified using the root mean square error (RMSE) and the mean absolute percent error (MAPE) relative to the known phantom values. Goodness-of-fit for regression models was assessed using the coefficient of determination ($R^{2}$). Statistical comparisons among predicted quantities were performed using the Kruskal–Wallis test with Dunn’s test for multiple comparisons. All analyses were conducted in GraphPad Prism (v9, GraphPad Software, Inc., San Diego, California, United States).

## 3 Results

### 3.1 Monte Carlo Simulation

#### 3.1.1 Dependence of $R_{0}^{\mathrm{MC}}$ on $\mu_{s}^{\prime }$ and g

**Fig. 2:**
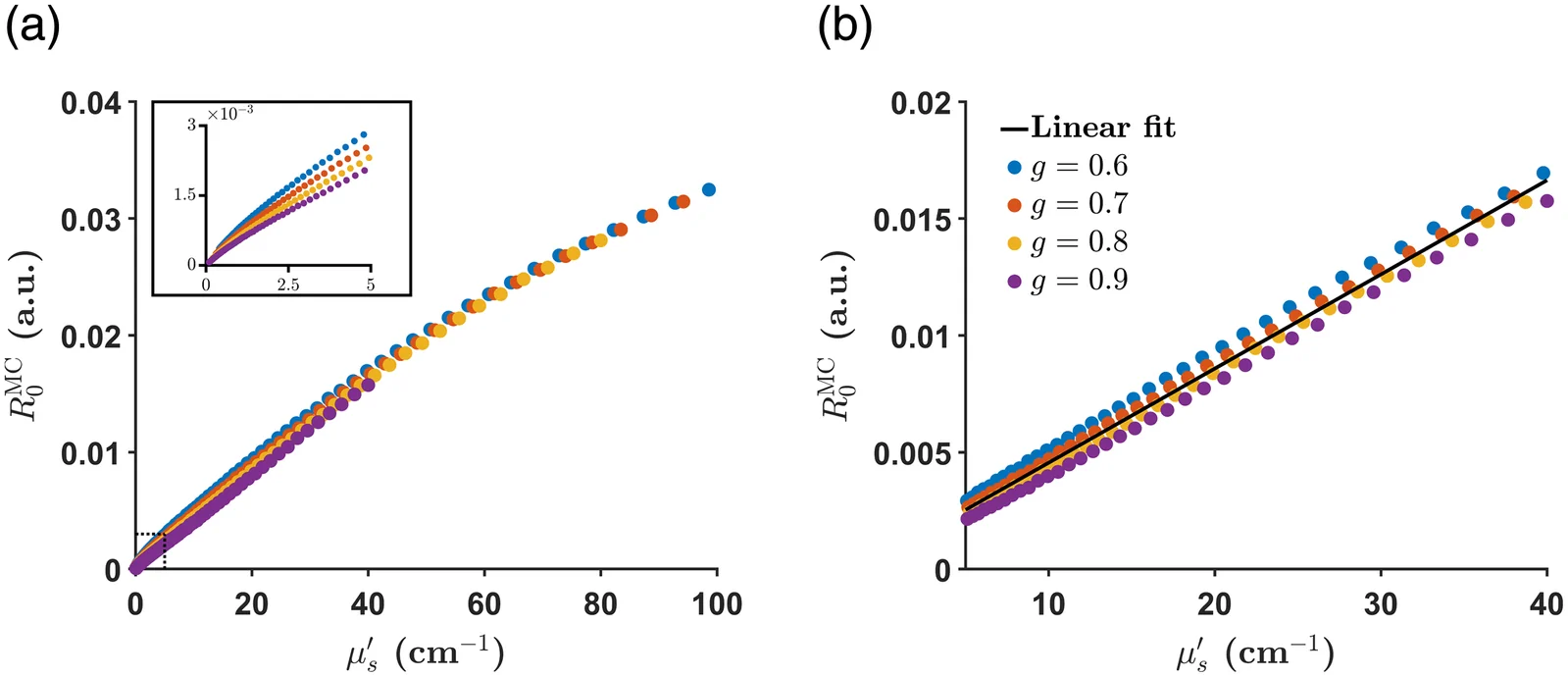
$R_{0}^{\mathrm{MC}}$ as a function of $\mu_{s}^{\prime }$ and g for (a) $\mu_{s}^{\prime }=0$ to $100\;{\mathrm{cm}}^{-1}$ and (b) $\mu_{s}^{\prime }=5$ to $35\;{\mathrm{cm}}^{-1}$. The dotted box in panel (a) indicates the low-scattering regime ($\mu_{s}^{\prime }<5\;{\mathrm{cm}}^{-1}$), enlarged in the inset. In both panels, circle color corresponds to the value of g, and the solid black line denotes the corresponding linear fit.

Figure 2(a) shows that the absorption-free scattering baseline ($R_{0}^{\mathrm{MC}}$) increases with increasing reduced scattering coefficient ($\mu_{s}^{\prime }$) up to a high $\mu_{s}^{\prime }$ value, beyond which it begins to saturate. The inset highlights the low-scattering regime ($\mu_{s}^{\prime }<5\;{\mathrm{cm}}^{-1}$), where the dependence becomes nonlinear and $R_{0}^{\mathrm{MC}}$ approaches zero as $\mu_{s}^{\prime }\to 0$. Figure 2(a) demonstrates that $R_{0}^{\mathrm{MC}}$ also depends on the scattering anisotropy g. With increasing g, scattering becomes more forward-directed, leading to a reduced $R_{0}^{\mathrm{MC}}$.

Although $R_{0}^{\mathrm{MC}}$ is g-dependent, we employ a g-independent model because g is not directly known or measurable *a priori* under experimental conditions. Figure 2(b) illustrates that within our region of interest ($5\;{\mathrm{cm}}^{-1}\leq \mu_{s}^{\prime }\leq 40\;{\mathrm{cm}}^{-1}$), $R_{0}^{\mathrm{MC}}$ can be expressed as a linear function of $\mu_{s}^{\prime }$ only, given by $R_{0}^{\mathrm{MC}}=4.0\times 10^{-4}\;\mu_{s}^{\prime }+5.3\times 10^{-4}$, with good agreement $\left(\mathrm{RMSE}=4.6\times 10^{-4},\;R^{2}=0.99\right)$. To reduce the number of independent parameters in the optical property extraction algorithm, $\mu_{s}^{\prime }$ was expressed as a linear function of $R_{0}^{\mathrm{MC}}$, given by $\mu_{s}^{\prime }=2449.87$ $R_{0}^{\mathrm{MC}}-1.07$ ($\mathrm{RMSE}=1.12\;{\mathrm{cm}}^{-1}$, $R^{2}=0.99$).

#### 3.1.2 Dependence of $\left\langle L_{\mathrm{SF}}^{\mathrm{MC}}\right\rangle$ on $\mu_{a}$, $\mu_{s}^{\prime }$, and g

**Fig. 3:**
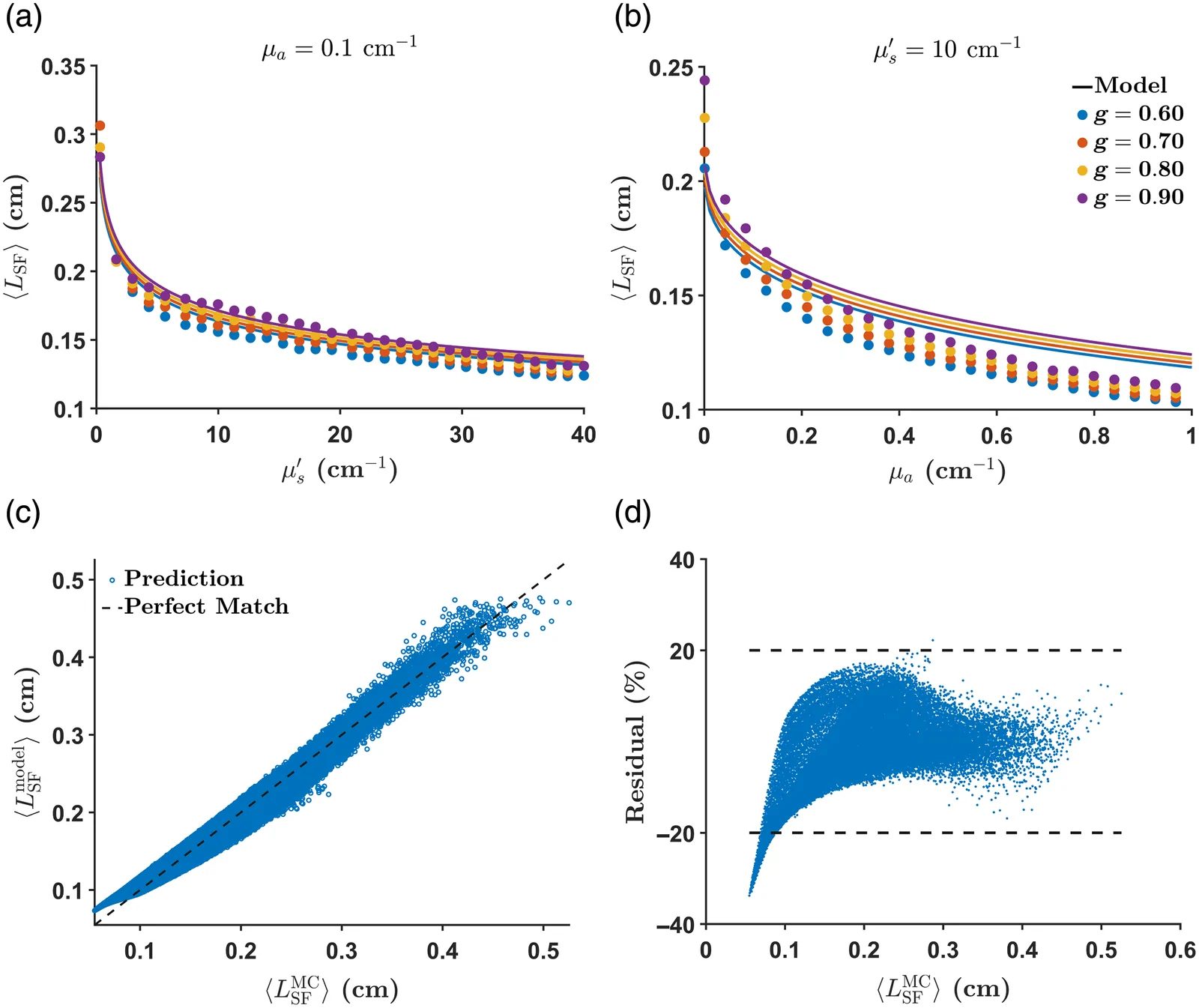
(a) $\left\langle L_{\mathrm{SF}}^{\mathrm{MC}}\right\rangle$ as a function of $\mu_{s}^{\prime }$ and g for fixed $\mu_{a}=0.1\;{\mathrm{cm}}^{-1}$. (b) $\left\langle L_{\mathrm{SF}}^{\mathrm{MC}}\right\rangle$ as a function of $\mu_{a}$ and g for fixed $\mu_{s}^{\prime }=10\;{\mathrm{cm}}^{-1}$. In panels (a) and (b), $\left\langle L_{\mathrm{SF}}^{\mathrm{MC}}\right\rangle$ is shown as circles and $\left\langle L_{\mathrm{SF}}^{\text{model}}\right\rangle$ as solid lines; different values of g are indicated by different colors. (c) Comparison between $\left\langle L_{\mathrm{SF}}^{\text{model}}\right\rangle$ and $\left\langle L_{\mathrm{SF}}^{\mathrm{MC}}\right\rangle$, with the dashed black line indicating perfect agreement. (d) Percent residual versus $\left\langle L_{\mathrm{SF}}^{\mathrm{MC}}\right\rangle$; horizontal dashed lines indicate ±20% residual error.

Figure 3(a) shows that the mean photon pathlength, $\left\langle L_{\mathrm{SF}}^{\mathrm{MC}}\right\rangle$, decreases with increasing $\mu_{s}^{\prime }$ for a fixed $\mu_{a}$ of $0.1\;{\mathrm{cm}}^{-1}$. For fixed $\mu_{a}$ and $\mu_{s}^{\prime }$, larger values of g correspond to increased $\left\langle L_{\mathrm{SF}}^{\mathrm{MC}}\right\rangle$. Figure 3(b) shows that $\left\langle L_{\mathrm{SF}}^{\mathrm{MC}}\right\rangle$ decreases with increasing $\mu_{a}$ for a fixed $\mu_{s}^{\prime }$ of $10\;{\mathrm{cm}}^{-1}$. The figure also illustrates the dependence on g: for fixed $\mu_{s}^{\prime }$ and $\mu_{a}$, $\left\langle L_{\mathrm{SF}}^{\mathrm{MC}}\right\rangle$ increases with increasing g.

Figures 3(a) and 3(b) also show that the semi-empirical mean photon pathlength model $\left\langle L_{\mathrm{SF}}^{\text{model}}\right\rangle$ described in Eq. (4) successfully captures the dependence of $\left\langle L_{\mathrm{SF}}^{\mathrm{MC}}\right\rangle$ on $\mu_{a}$, $\mu_{s}^{\prime }$, and g. Although some mismatch is observed between $\left\langle L_{\mathrm{SF}}^{\mathrm{MC}}\right\rangle$ and $\left\langle L_{\mathrm{SF}}^{\text{Model}}\right\rangle$, particularly in Fig. 3(b) due to the limited flexibility of $\left\langle L_{\mathrm{SF}}^{\text{model}}\right\rangle$, the general agreement is strong. This agreement is further demonstrated in Fig. 3(c), which compares $\left\langle L_{\mathrm{SF}}^{\mathrm{MC}}\right\rangle$ and $\left\langle L_{\mathrm{SF}}^{\text{model}}\right\rangle$ across the full parameter space. The model $\left\langle L_{\mathrm{SF}}^{\text{model}}\right\rangle$ provides an excellent fit to $\left\langle L_{\mathrm{SF}}^{\mathrm{MC}}\right\rangle$ (RMSE=0.012  cm, MAPE=6.2%, $R^{2}=0.975$), resulting in the following fitted parameters: $p_{1}=2.98486$, $p_{2}=0.51711$, $p_{3}=0.18911$, $p_{4}=4.37162\times 10^{-7}$, $p_{5}=2.49478$, and $p_{6}=0.471541$. These fitted parameters were used without modification for all subsequent tissue optical property extraction from experimental data. The residual plot in Fig. 3(d) shows that, for most pathlengths, the percentage error remains below 20%, with larger deviations occurring primarily at very small pathlengths.

#### 3.1.3 Dependence of the number of scattering events on $\mu_{s}^{\prime }$ and g

**Fig. 4:**
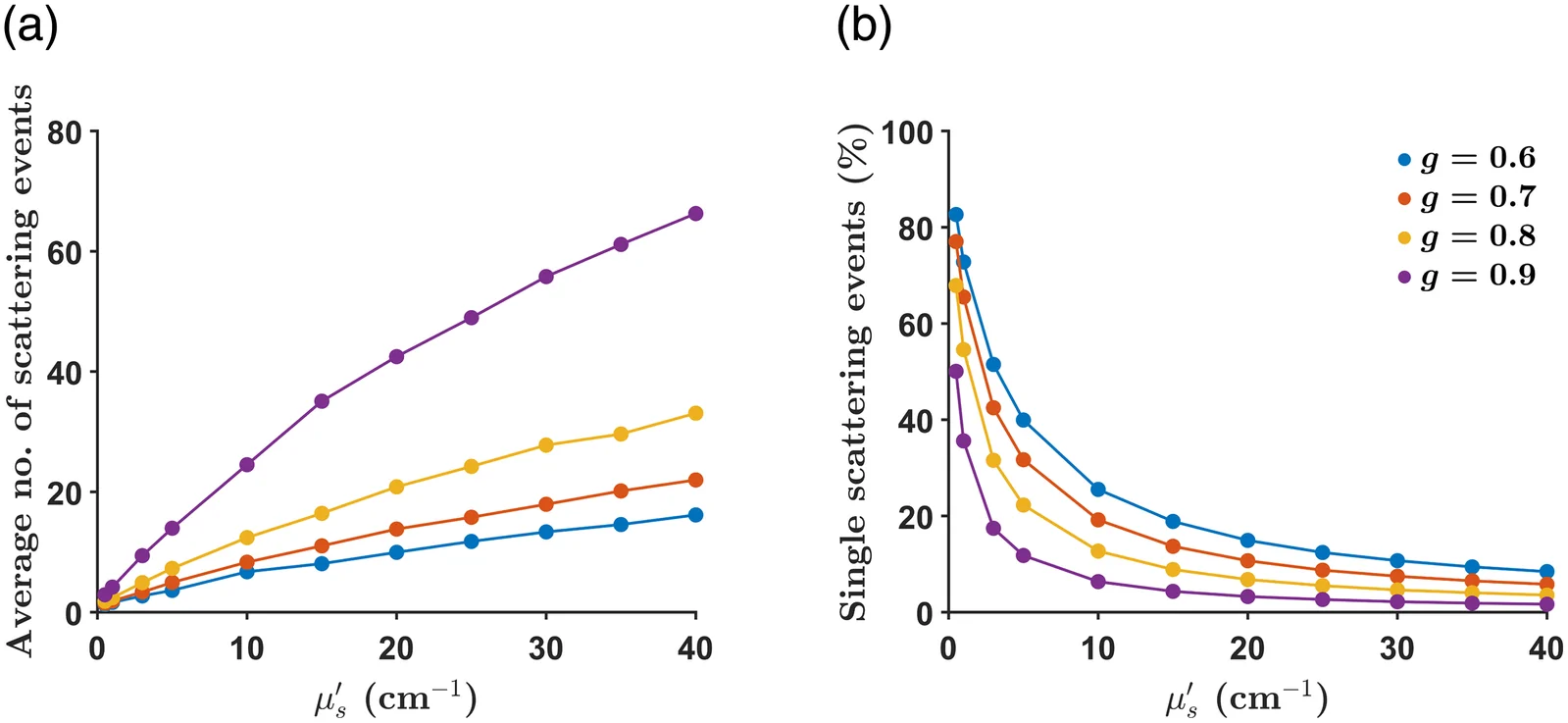
Dependence of scattering events on $\mu_{s}^{\prime }$ and g for MC simulation with a fixed $\mu_{a}=0.001\;{\mathrm{cm}}^{-1}$. (a) Average number of scattering events experienced by a photon packet prior to detection as a function of $\mu_{s}^{\prime }$ for different values of g. (b) Percentage of detected photon packets undergoing a single scattering event as a function of $\mu_{s}^{\prime }$ for different values of g. In both panels, circle color corresponds to the value of g.

Figure 4(a) shows that, for a fixed $\mu_{a}=0.001\;{\mathrm{cm}}^{-1}$, the average number of scattering events experienced by a photon packet before detection increases with increasing $\mu_{s}^{\prime }$ and g. Figure 4(b) shows that, for a fixed $\mu_{a}=0.001\;{\mathrm{cm}}^{-1}$, in the low-scattering regime ($\mu_{s}^{\prime }<5\;{\mathrm{cm}}^{-1}$), most photon packets experience a single scattering event before detection. Therefore, the SFR signal is dominated by single scattering events. As $\mu_{s}^{\prime }$ increases, a larger fraction of photon packets undergo multiple scattering events, leading to increased contributions from multiply scattered photons to the overall SFR signal. The figure also shows that decreasing g enhances the relative contribution of single-scattering events.

### 3.2 Experimental SFR Spectra and Fitting: Contribution of Single-Scattered and Multiply Scattered Photons

The overall SFR spectra consist of contributions from both single- and multiply scattered photons, as well as specular reflection originating from the fiber–sample interface. The calibration procedure described in Sec. 2.3 eliminates most of the specular reflection. The relative contributions of singly back-scattered and multiply scattered photons depend on the optical properties of the sample, as shown in Sec. 3.1.3. These contributions also depend on fiber parameters such as the fiber NA and diameter.

**Fig. 5:**
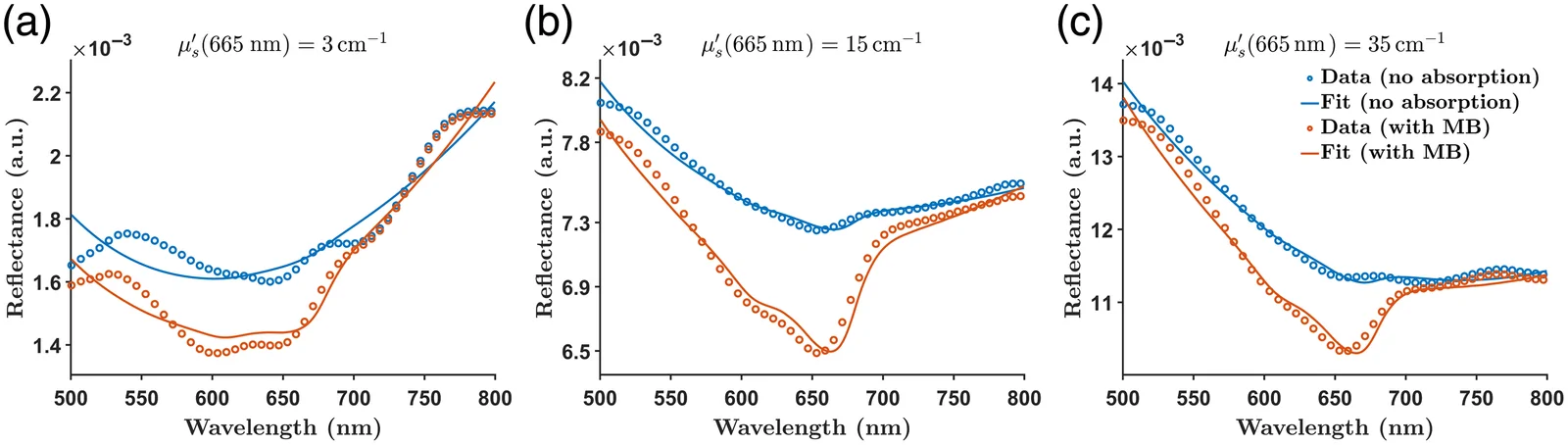
Experimental SFR spectra (open circles) and corresponding fits (solid lines) for phantoms with $\mu_{s}^{\prime }(665\;\mathrm{nm})$ of (a) 3, (b) 10, and (c) $35\;{\mathrm{cm}}^{-1}$, shown without absorber (blue) and with MB at $C_{\mathrm{MB}}=6.7\;\mu M$ (orange).

Figures 5(a), 5(b), and 5(c) show how both the magnitude and the spectral shape of the experimental SFR signal evolve from the low- to intermediate- to high-scattering regimes. In the low-scattering regime [phantom with $\mu_{s}^{\prime }(665\;\mathrm{nm})=3\;{\mathrm{cm}}^{-1}$; Fig. 5(a)], single-scattering events dominate the detected SFR signal, as shown previously in Fig. 4(b), and the spectrum does not follow a simple decaying power law relationship. For the intermediate-scattering phantom [$\mu_{s}^{\prime }(665\;\mathrm{nm})=15\;{\mathrm{cm}}^{-1}$; Fig. 5(b)], the relative contribution of singly scattered photons decreases while the contribution from multiply scattered photons increases, as demonstrated in Fig. 4(b). At high scattering [phantom with $\mu_{s}^{\prime }(665\;\mathrm{nm})=35\;{\mathrm{cm}}^{-1}$; Fig. 5(c)], the SFR signal is dominated by multiply scattered (diffuse) contributions, and the SFR spectrum follows the decaying power-law behavior expected for diffuse reflectance.^26^

To account for contributions from both singly scattered and multiply scattered photons, $R_{0}(\lambda )$ was parameterized as the sum of two power law terms representing singly scattered (sub-diffusive) and multiply scattered (diffuse) contributions, as described in Eq. (5). Figures 5(a), 5(b), and 5(c) also demonstrate that this parameterization of $R_{0}(\lambda )$, together with the semi-empirical mean pathlength model, leads to agreement between the experimental and modeled SFR spectra for phantoms, both with and without absorption, across a wide range of optical properties.

### 3.3 Optical Property Recovery

#### 3.3.1 Performance of fiber 1

**Fig. 6:**
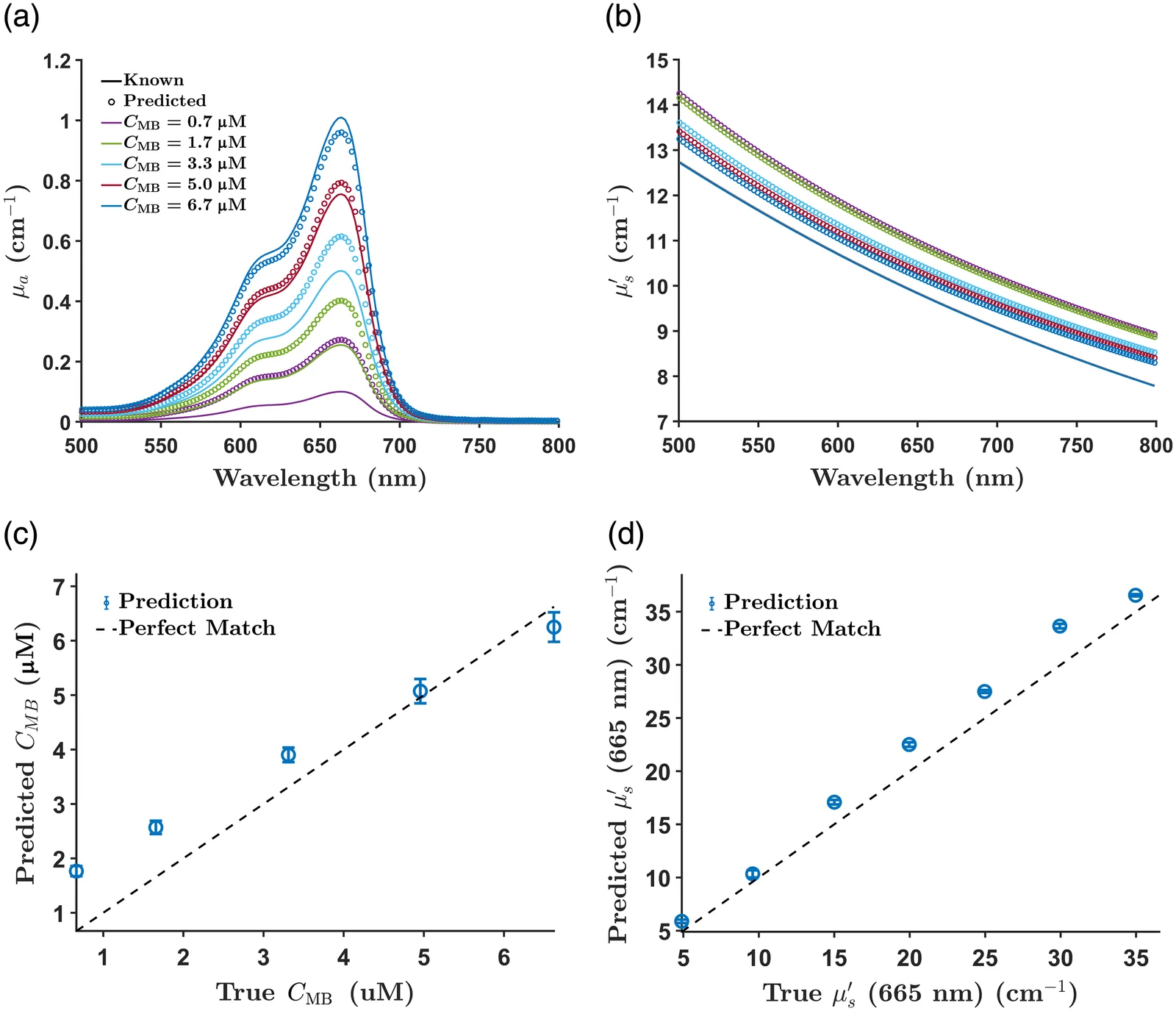
Recovered (a) $\mu_{a}(\lambda )$ and (b) $\mu_{s}^{\prime }(\lambda )$ for a representative phantom with $\mu_{s}^{\prime }(665\;\mathrm{nm})=9.6\;{\mathrm{cm}}^{-1}$. The known spectra are represented by solid lines, and the predicted spectra by open circles, with colors indicating the MB concentration. (c) Recovered $C_{\mathrm{MB}}$ and (d) $\mu_{s}^{\prime }(665\;\mathrm{nm})$ across all phantoms. Predicted values are marked by blue open circles, and the dashed black line denotes the perfect agreement. Each symbol represents the mean recovered value from all phantom measurements with identical optical properties, and error bars correspond to the standard deviation across those phantoms. Error bars are included in all cases but may not always be visible.

The accuracy of optical property recovery obtained from the validation phantom dataset ($V_{F1}$) using fiber 1 is summarized in Fig. 6. Representative recovered absorption and reduced scattering spectra corresponding to a phantom with $\mu_{s}^{\prime }=9.6\;{\mathrm{cm}}^{-1}$ at 665 nm and $\mu_{a}$ values of 0.1, 0.25, 0.5, 0.75, and $1.0\;{\mathrm{cm}}^{-1}$ at 665 nm are shown in Figs. 6(a) and 6(b), respectively. These results demonstrate the agreement between recovered spectra and ground truth across the full wavelength range.

MB concentration ($C_{\mathrm{MB}}$) in the validation phantoms was recovered with an RMSE of 0.73   μM (MAPE=49.8%), as shown in Fig. 6(c). The higher MAPE in MB concentration recovery arises primarily from low-$C_{\mathrm{MB}}$ phantoms, for which minor absolute deviations lead to large percentage errors. When restricted to $C_{\mathrm{MB}}>1\;\mu M$, MB recovery yielded an RMSE of 0.61  μM (MAPE=20.8%). At 665 nm, the reduced scattering coefficient $\mu_{s}^{\prime }(665\;\mathrm{nm})$ was recovered with an RMSE of $2.23\;{\mathrm{cm}}^{-1}$ (MAPE=11.6%), as shown in Fig. 6(d). Across all wavelengths, the reduced scattering coefficient $\mu_{s}^{\prime }(\lambda )$ was recovered with an RMSE of $2.36\;{\mathrm{cm}}^{-1}$ (MAPE=12%).

#### 3.3.2 Performance across fibers

**Fig. 7:**
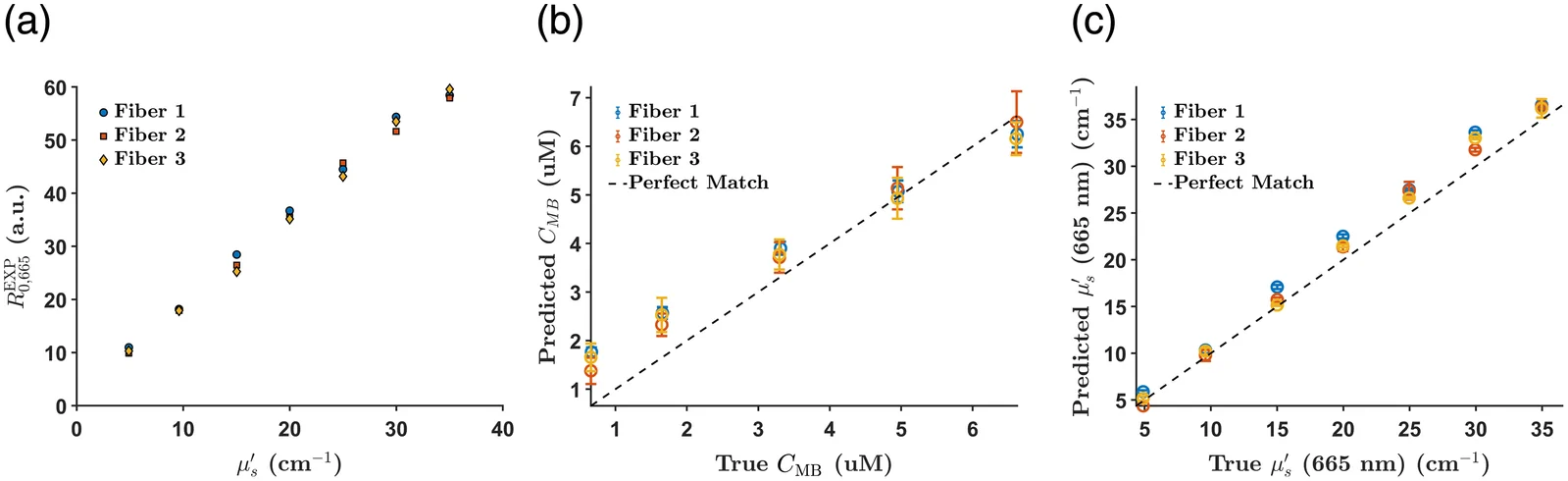
(a) Calibrated SFR signal at 665 nm versus $\mu_{s}^{\prime }(665\;\mathrm{nm})$ for all validation phantoms, with different colors and symbols indicating different fibers. (b) Recovered $C_{\mathrm{MB}}$ versus known values. (c) Recovered $\mu_{s}^{\prime }(665\;\mathrm{nm})$ versus known values. In panels (b) and (c), different fibers are shown in different colors, and the dashed black line denotes perfect agreement. Error bars represent the standard deviation across phantoms with identical optical properties and are included in all cases, though they may not always be visible.

The calibration procedure described in Sec. 2.3 reduces the differences among experimental SFR signals acquired using individual optical fibers that arise from manufacturing variability, such as fiber throughput. After calibration, the mean percentage absolute deviation (MPAD) between the experimental signals acquired using fibers 1 and 2 was 8%, while the MPAD between fibers 2 and 3 was 4.8%. Figure 7(a) shows the calibrated SFR data at 665 nm $R_{0,665}^{\mathrm{EXP}}$ versus $\mu_{s}^{\prime }(665\;\mathrm{nm})$ for all validation phantoms for different fibers, demonstrating minimal inter-fiber differences in the calibrated signals. This calibration therefore enables the use of a single recovery model across multiple fibers.

The optical property recovery results for the validation datasets $V_{F2}$ and $V_{F3}$, collected using fibers 2 and 3, are shown in Figs. 7(b) and 7(c). MB concentration ($C_{\mathrm{MB}}$) was recovered with an RMSE of 0.61  μM (MAPE=35.7%) for fiber 2 and 0.74  μM (MAPE=46.8%) for fiber 3. When restricted to $C_{\mathrm{MB}}>1\;\mu M$, MB recovery yielded an RMSE of 0.57  μM (MAPE=17%) for fiber 2 and 0.64  μM (MAPE=20.3%) for fiber 3. The reduced scattering coefficient at 665 nm, $\mu_{s}^{\prime }(665\;\mathrm{nm})$, was recovered with an RMSE of $1.48\;{\mathrm{cm}}^{-1}$ (MAPE=7.1%) for fiber 2 and $1.58\;{\mathrm{cm}}^{-1}$ (MAPE=5.9%) for fiber 3. Across all wavelengths, the reduced scattering coefficient $\mu_{s}^{\prime }(\lambda )$ was recovered with an RMSE of $1.58\;{\mathrm{cm}}^{-1}$ (MAPE=7.3%) for fiber 2 and $1.68\;{\mathrm{cm}}^{-1}$ (MAPE=6.3%) for fiber 3. The $C_{\mathrm{MB}}$ recovery results for fibers 2 and 3 are comparable to those for fiber 1 in terms of accuracy but exhibit a slightly higher spread across different scattering conditions. In contrast, the recovery of $\mu_{s}^{\prime }(665\;\mathrm{nm})$ is comparatively better. A Kruskal–Wallis test followed by Dunn’s multiple comparisons test showed no significant differences among fibers for recovered $C_{\mathrm{MB}}$ (p=0.88) or $\mu_{s}^{\prime }(665\;\mathrm{nm})$ (p=0.68).

## 4 Discussion and Conclusion

In this work, we demonstrated that an unmodified, FDA-approved clinical optical fiber can be used in an SFR spectroscopy system to recover tissue optical properties. We employed a semi-empirical model that relates the SFR spectrum to the underlying absorption and reduced scattering coefficients through an explicit description of the mean photon pathlength, informed by MC simulations of light transport in the single-fiber geometry. To reduce the number of free parameters in the optical property inversion problem, absorption and scattering were parameterized. The absorbers present were assumed to be known *a priori*, while scattering was modeled using a wavelength-dependent power law relationship. Using this framework, the recovery of optical properties was achieved from experimental SFR measurements collected from tissue-simulating phantoms. The same model achieved comparable accuracy in predicting optical properties for nominally identical fibers without the need for model adaptation.

Our SFR spectroscopy system is based on an unmodified, single clinical fiber that costs substantially less than the custom multi-fiber probes used in our previous systems ($600 versus $3000).^12^ Because this fiber is commercially manufactured rather than custom-built, it can be readily acquired in large quantities with minimal lead time. Importantly, the fiber is used in its unmodified form, which avoids the safety concerns associated with angle-polished or custom-fabricated probes and simplifies regulatory approval. As a sterile, single-use clinical fiber, it can be used directly out of the package for each patient, eliminating the need for elaborate sterilization procedures. In addition, the clinical fiber can withstand high laser powers, enabling the same fiber to be used for both optical property recovery and subsequent PDT light delivery. Because these fibers are manufactured at scale, fiber-to-fiber variability is reduced, and we demonstrate that the same inversion model can be applied across nominally identical fibers with comparable accuracy. This removes the need for time- and resource-intensive probe-specific calibration or model redevelopment,^14^^,^^15^ facilitating routine use of a new fiber for each procedure. Together with the simplified optical instrumentation and compact system footprint, these features make SFR spectroscopy using unmodified clinical fibers particularly compelling for scalable clinical deployment to support our PDT treatment planning in large patient cohorts.

We are specifically interested in optical property recovery to inform treatment planning for PDT of abdominopelvic abscesses.^4^^,^^13^ Building on the success of our phase 1 clinical trial,^4^ we are advancing toward a phase 2 trial with the longer-term goal of multi-center studies and widespread clinical adoption. From this translational perspective, SFR spectroscopy is particularly well suited, as the characteristics of our clinical problem align closely with those of SFR spectroscopy. For our PDT treatment planning, the objective is to achieve a prescribed target fluence rate at the abscess wall. Previously, we showed that, for a representative abscess geometry, achieving less than 20% error in the recovery of $\mu_{a}$ results in less than 10% error in the prescribed optical power.^12^ This criterion is satisfied for absorption coefficients in the range ($\mu_{a}(665\;\mathrm{nm})=0.25$ to $1\;{\mathrm{cm}}^{-1}$). In the low-absorption regime, where $\mu_{a}<0.25\;{\mathrm{cm}}^{-1}$, small absolute errors lead to large percentage errors exceeding 20%. Future work will focus on achieving clinically sufficient accuracy over the full range of absorption coefficients observed in abscess cavities ($\mu_{a}(665\;\mathrm{nm})=0.1-5\;{\mathrm{cm}}^{-1}$), as discussed below.

Several prior studies have explored optical property recovery with SFR spectroscopy. Using a semi-empirical model in a multi-diameter SFR configuration, Kanick et al. demonstrated recovery of $\mu_{s}^{\prime }$ and the phase-function parameter γ with accuracies of 5% and 3%, respectively, across a wide range of scattering conditions in MC studies,^27^ with subsequent tissue-mimicking phantom experiments yielding mean errors of 5.5% for $\mu_{s}^{\prime }$ and 4.8% for γ.^28^ Additional MC studies showed that $\mu_{a}$ could be recovered with average errors below 7.5%.^29^ Using an alternative model^30^ incorporating the phase-function parameter $p_{\mathrm{sb}}$, Post et al. demonstrated optical property recovery in tissue-mimicking phantoms. When implemented with a single-fiber, the average recovery errors were ∼33% for $\mu_{a}$, 50% for $\mu_{s}^{\prime }$, and 186% for $p_{\mathrm{sb}}$.^17^ Compared with these previous results, our system exhibited similar $\mu_{s}^{\prime }$ recovery performance but lower $\mu_{a}$ recovery accuracy. However, these prior studies relied on the multi-diameter single-fiber approach with an angle-polished distal end, whereas our system uses an unmodified, flat-cleaved single clinical fiber to satisfy our aforementioned clinical constraints. In this work, we prioritized simplicity and clinical scalability over maximizing recovery accuracy. In contrast to multi-fiber and multi-diameter approaches that aim to recover a larger number of optical parameters, our approach restricts the inversion to the parameters most relevant for PDT treatment planning, namely, $\mu_{a}$ and $\mu_{s}^{\prime }$.^6^^,^^11^ Compared with multi-fiber systems,^31^^–^^34^ our SFR spectroscopy system exhibited somewhat lower $\mu_{a}$ recovery accuracy, particularly in low-absorption regimes. This behavior is expected, as the collocated illumination and collection geometry in SFR lead to shorter photon pathlengths and reduced sensitivity to absorption.^15^

An important consideration when using an unmodified, flat-cleaved single fiber is the removal of specular reflection at the fiber–sample interface. In phantom measurements, specular reflection was addressed through subtraction of a water reference spectrum (see Eq. 2). Because the refractive index of the phantoms remains close to that of water over the concentration range studied, water reference subtraction provides a reasonable correction for removing specular reflection. For *in vivo* tissue measurements, where the refractive index is higher (typically $n_{\text{tissue}}\approx 1.37$),^10^ accurate removal of specular reflection requires either an index-matched calibration measurement or explicit modeling of refractive index mismatch effects.

Although this study demonstrates optical property recovery with clinically useful accuracy using SFR spectroscopy, several limitations must be considered to advance toward robust clinical implementation. The MC simulations used to develop the semi-empirical SFR model were performed using only the Henyey–Greenstein phase function, which is commonly used to represent tissue scattering, although other phase functions have also been shown to better describe tissue scattering behavior in certain contexts.^15^ A mismatch between the phase function assumed during model development and the actual tissue scattering phase function may bias the recovered reduced scattering coefficient because the angular distribution of scattered photons collected by the fiber would differ from that predicted by the model. Consequently, the optical property extraction algorithm may compensate for this discrepancy by overestimating or underestimating the reduced scattering coefficient. In future work, a broader range of tissue-relevant phase functions will be incorporated into MC simulations, and their impact on optical property recovery accuracy will be evaluated. Furthermore, experimental validation was limited to a representative absorber (MB) and scatterer (Intralipid-20%), within a biologically relevant but restricted range of optical properties ($\mu_{a}(665\;\mathrm{nm})=0.1$ to $1\;{\mathrm{cm}}^{-1}$ and $\mu_{s}^{\prime }(665\;\mathrm{nm})=5$ to $35\;{\mathrm{cm}}^{-1}$). MB was selected as the representative absorber because it is the photosensitizer used in our PDT treatment. Although this framework can be extended to other absorbers and scatterers, the performance of the algorithm in the presence of multiple absorbers has not yet been established. The objective of this study was to demonstrate accurate recovery of optical properties for a representative absorber and scatterer, rather than evaluate system performance under complex tissue conditions containing multiple endogenous absorbers such as oxyhemoglobin and deoxyhemoglobin. In our prior phase 1 clinical trial, MB, oxyhemoglobin, and deoxyhemoglobin were identified as the dominant absorbers in abscess cavities,^13^ motivating future phantom studies that incorporate multiple absorbers across the full physiologically relevant optical property range observed in abscesses ($\mu_{a}(665\;\mathrm{nm})=0.1$ to $5\;{\mathrm{cm}}^{-1}$ and $\mu_{s}^{\prime }(665\;\mathrm{nm})=2.5$ to $35\;{\mathrm{cm}}^{-1}$). Finally, although the semi-empirical model employed in this study offers physical interpretability, its limited flexibility leads to reduced performance in low-absorption and low-scattering regimes. Future work will explore physics-informed neural networks^35^ and hybrid analytical ML architectures^36^^,^^37^ to enhance system performance while balancing interpretability and flexibility, thereby overcoming the limitations of purely analytical or purely data-driven approaches.

This work presents a proof-of-concept for the translational potential of an SFR spectroscopy system with an unmodified, FDA-approved clinical optical fiber for estimating tissue optical properties. Moving forward, we will continue to develop SFR spectroscopy as a robust method for determining tissue optical properties in clinical environments, with a focus on supporting PDT treatment planning.

## Data Availability

The data underlying the results presented in this paper are not publicly available at this time but may be obtained from the authors upon reasonable request.
